# Haemophagocytic lymphohistiocytosis in patients with human immunodeficiency virus infection: to treat or not to treat

**DOI:** 10.11604/pamj.2019.32.105.14764

**Published:** 2019-03-06

**Authors:** Farhan Fazal, Nitin Gupta, Shivabalan Kathavarayan Ramu, Aditya Nayan, Naval Kishore Vikram, Animesh Ray

**Affiliations:** 1 Department of Medicine, All India Institute of Medical Sciences, New Delhi, India

**Keywords:** Malignancy, steroids, hospital acquired pneumonia

## Abstract

Haemophagocytic lymphohistiocytosis (HLH) in Human Immunodeficiency Virus (HIV) infected individuals can either be due to the disease itself or due to associated infections/malignancies. The treatment for HLH requires immunosuppressive therapy but administering immunosuppressive therapy to an already immunosuppressed patient (HIV infection) is complex. We present two such cases of HLH in patients infected with HIV. In the first case, no alternate cause for HLH was found even after extensive investigations and it was attributed to the uncontrolled HIV replication. Patient was started on dexamethasone for the same but succumbed to hospital acquired pneumonia. The second patient was diagnosed with Hodgkin's lymphoma but he succumbed to his illness before initiating immunosuppressive therapy for HLH. We report these cases to highlight the dilemma and a need for further research in this direction.

## Introduction

Haemophagocytic lymphohistiocytosis (HLH) is an aggressive syndrome of excessive immune activation. It is diagnosed using the HLH- 2004 protocol which requires fulfilling five out of eight criteria (fever, splenomegaly, bicytopenia, hypertriglyceridemia and/or low fibrinogen, hyperferritinemia, increased soluble CD25 levels, decreased Natural killer (NK) cell activity and histopathological evidence of hemophagocytosis [1]. Out of these, NK cell activity and serum CD25 levels are not widely available, making the diagnosis of HLH often difficult. This problem becomes more complex in patients with Human Immunodeficiency Virus (HIV) infection as features such as fever, bicytopenia and splenomegaly are already common in these patients with or without presence of opportunistic infections. Once diagnosed, the treatment of such a patient is even more challenging. The biggest hurdle is the use of immunosuppression for the treatment of HLH in an already immunocompromised person. A balance has to be struck between controlling inflammation with immunosuppressive drugs and limiting the risk of life threatening opportunistic infections. We present two cases of HIV with HLH to explain our dilemma.

## Patient and observation

**Case 1**: a 38-year old female patient, diagnosed with HIV infection in 2008, presented with complaints of intermittent high grade fever associated with chills and rigor for one month to a local hospital. This was associated with loss of appetite and generalized weakness. She was transfused two units of packed RBC. She was receiving an antiretroviral regimen consisting of tenofovir, lamivudine and efavirenz. Her CD4 count was 85/μl and the viral load was 56, 670 copies/μl. With a diagnosis of virological failure, she was shifted to an atazanavir/ritonavir based regimen. She was referred to us with persistent fever. On examination, she was febrile with a pulse rate of 120/min and a respiratory rate of 25/min. She had icterus and her jugular venous pressure was elevated. Chest examination revealed decreased bilateral breath sounds and bi-basal crepitations. On abdominal examination hepatosplenomegaly was present. The baseline laboratory evaluation revealed pancytopenia and hyperbilirubinemia (Hemoglobin- 5.9 gm/dl, total leucocyte count- 1500/cu.mm, platelet count- 18,000/cu.mm and bilirubin- 3.3gm/dl). Peripheral smear showed dimorphic hypochromic anemia with a corrected reticulocyte count of 1%. Vitamin B12 and folic acid levels were normal. Lactate dehydrogenase (LDH) levels were elevated (1154 U/l). Blood culture was sterile for bacteria, fungi and non-tubercular mycobacteria. Contrast enhanced computed tomography (CECT) scan of chest and abdomen revealed hepatosplenomegaly (liver-16.8 cm, spleen-13.4cm) and multiple enlarged non-necrotic lymph nodes in mesentery, para-aortic and inguinal region. A whole body Fluorodeoxy glucose positron emission tomography (FDGPET) scan revealed hypermetabolic bilateral supraclavicular, internal mammary lymph nodes and abdominal lymph nodes. There was avid uptake in liver, spleen and bone marrow also. The biopsy from supraclavicular lymph node showed reactive hyperplasia. Staining for acid fast bacilli, GeneXpert and Mycobacterial growth indicator tube (MGIT) culture for Mycobacterium tuberculosis were negative. A bone marrow biopsy was done which showed 60-70% cellularity. It was negative for geneXpert and Cytomegalovirus (CMV) polymerase chain reaction (PCR) assay. Also, pp65 antigen detection test in blood for CMV and rk39-antibody test for visceral leishmaniasis was negative. With a presumptive diagnosis of tuberculosis, modified anti-tubercular therapy (ATT) (levofloxacin, ethambutol and amikacin) was started as the patient had elevated bilirubin level. There was no response even after one month of ATT. Introduction of rifampicin and isoniazid was attempted but the bilirubin levels rose to 9.5g/dl. Clarithromycin was empirically added to cover for Mycobacterium avium complex (MAC) infection. On further investigations, she was found to have a triglyceride levels of 435 mg/dl, fibrinogen levels of 500 mg/dl, ferritin levels of >2000 ng/ml and decreased NK cell activity. With a diagnosis of Haemophagocytic lymphohistiocytosis (HLH), dexamethasone at a dose of 16 mg per day was started. The fever and pancytopenia improved in a week's time (Hemoglobin- 7.4gm/dl, total leucocyte count- 5300/cu.mm, platelet count- 50,000/cu.mm). The patient was doing well but she started getting dyspneic fifteen days after the initiation of steroids. Chest X-ray revealed consolidation in the right middle lobe. With a diagnosis of hospital acquired pneumonia, she was started on cefoperazone sulbactam, but she succumbed to her illness after two days.

**Case 2**: a 46-year old male patient on tenofovir, lamivudine and efavirenz, presented with intermittent low grade fever for the last four months. This was associated with night sweats, loss of appetite and loss of weight of around five kilograms. He also complained of decrease in urine output and generalized swelling of the body. On general examination, he was febrile and was found to have enlarged right axillary lymph node (1cm x 1cm). On systemic examination, he had ascites and a palpable spleen (8 cm below the left costal margin). Fundus examination was normal. On laboratory investigations, he was found to have pancytopenia, deranged liver function and kidney function tests (Hemoglobin- 7.4g/dl, total leucocyte count-1200/mcl, platelet count-20000/mcl, aspartate transaminase/alanine transaminase-209/117 U/l and urea/creatinine- 78/1.7 mg/dl). His baseline CD4 was 221/μl and the most recent CD4 was 158/μl. Non contrast computed tomography of abdomen revealed multiple enlarged retroperitoneal lymph nodes with the largest measuring 47 x 22 mm. Lymph node biopsy could not be performed due to deranged coagulation parameters. Blood and urine cultures were sterile. Peripheral smear showed normocytic normochromic anemia. Vitamin B12 levels were normal but the folate levels were low (2.2ng/ml). Serum LDH levels were elevated (834 IU/l). Immunochromatography for rk39 antibody was negative. Ascitic fluid analysis revealed a protein of 1.9 g/dl, albumin of 0.9 g/dl, total leucocyte counts of 380/mcl (Lymphocytes 90%, Neutrophils 10%), serum-ascitic albumin gradient of 1.1g/dl and adenosine deaminase levels of 40 IU/l. Ascitic fluid cultures were sterile. With a presumptive diagnosis of disseminated tuberculosis, he was started on ATT. His ferritin levels were elevated (>2000 ng/ml) and triglyceride levels were also high (324 mg/dl). A presumptive diagnosis of HLH was made. A lymph node biopsy was performed after correction of coagulation abnormalities to identify the primary pathology. However, he succumbed to his illness before the results of biopsy were available. The biopsy was suggestive of Hodgkin's lymphoma.

## Discussion

Although, there are studies where hemophagocytosis has been shown in patients with HIV, reports of HIV with HLH meeting the 2004 criteria are rare [2]. These reports are even more scarce from the Indian subcontinent. A study by Kotwal *et al*. showed hemophagocytic picture in eight patients with HIV and haematological abnormalities but only two patients fulfilled the HLH diagnostic criteria [3]. Isolated case reports of HIV patients with leishmaniasis or histoplasmosis who developed HLH has been reported [4, 5]. HLH in HIV patient can either be due to the disease itself or because of the opportunistic infections or malignancy associated with HIV [6, 7]. In the first patient, we extensively looked for an opportunistic infection that could have caused the secondary HLH but did not find any. The first patient was treated with steroids and she did show improvement initially. But in the process of treatment, with further immunosuppression, she became prone to the hospital acquired infections and succumbed to pneumonia. The likely triggering factor in the first patient was uncontrolled HIV replication itself as she was failing on the regimen she was receiving. In a report by Kyung *et al.*, HLH in a patient with acute HIV could not be attributed to any infection/malignancy except for HIV itself [8]. That HIV alone can cause HLH is further proven by the response to Highly Active Antiretroviral Therapy (HAART) in patients with HLH and acute HIV [9]. In the second patient, more conservative approach to initiation of steroids was taken but the patient succumbed to the primary disease which was later confirmed as Hodgkin's lymphoma. AIDS defining malignancy was the likely etiology for secondary HLH in this patient. Although, HLH, lymphoma and HIV are a rare combination, few reports are available in the published literature [6]. Since both HIV and lymphoma promote cytokine release, the likelihood of occurrence of secondary HLH in such cases increases. The treatment of HLH requires aggressive chemoimmunotherapy including steroids, etoposide and cyclosporin A. However, there is a lack of evidence regarding utility of immunosuppressants in patients with HIV who develop HLH. In a retrospective study of 58 patients with HIV and HLH by Fardet *et al.*, there was no significant difference in survival between those who received HLH specific treatment and those who did not [10].

## Conclusion

We report these cases to raise a pertinent question regarding the management of HLH in patients with HIV. Although, there is no dearth in availability of triggers in patients with HIV, there is a definite gap in the literature regarding their management. Due to the variability in the nature of these triggers, development of a standard therapeutic protocol is difficult. There is a definite need for further research in this area.

## Competing interests

The authors declare no competing interests.
